# *Trichoderma erinaceum* Bio-Priming Modulates the WRKYs Defense Programming in Tomato Against the *Fusarium oxysporum* f. sp. *lycopersici* (*Fol*) Challenged Condition

**DOI:** 10.3389/fpls.2019.00911

**Published:** 2019-07-30

**Authors:** Mohd Aamir, Sarvesh Pratap Kashyap, Andleeb Zehra, Manish Kumar Dubey, Vinay Kumar Singh, Waquar Akhtar Ansari, Ram S. Upadhyay, Surendra Singh

**Affiliations:** ^1^Laboratory of Mycopathology and Microbial Technology, Centre of Advanced Study in Botany, Institute of Science, Banaras Hindu University, Varanasi, India; ^2^Division of Crop Improvement and Biotechnology, Indian Institute of Vegetable Research, Indian Council of Agricultural Research, Varanasi, India; ^3^Department of Botany, Institute of Science, Banaras Hindu University, Varanasi, India; ^4^Centre for Bioinformatics, School of Biotechnology, Institute of Science, Banaras Hindu University, Varanasi, India

**Keywords:** bio-priming, defense transcriptome, *WRKY* genes, lignification, gene expression

## Abstract

The beneficial association and interaction of rhizocompetent microorganisms are widely used for plant biofertilization and amelioration of stress-induced damage in plants. To explore the regulatory mechanism involved in plant defense while associating with beneficial microbial species, and their interplay when co-inoculated with pathogens, we evaluated the response of tomato defense-related *WRKY* gene transcripts. The present study was carried out to examine the qRT–PCR-based relative quantification of differentially expressed defense-related genes in tomato (*Solanum lycopersicum* L.; variety S-22) primed with *Trichoderma erinaceum* against the vascular wilt pathogen (*Fusarium oxysporum* f. sp. *lycopersici*). The tissue-specific and time-bound expression profile changes under the four different treatments “(unprimed, *Fol* challenged, *T. erinaceum* primed and *Fol*+ *T. erinaceum*)” revealed that the highest upregulation was observed in the transcript profile of *SlWRKY31* (root) and *SlWRKY37* (leaf) in *T. erinaceum* bioprimed treated plants at 24 h with 16.51- and 14.07-fold increase, respectively. In contrast, *SlWRKY4* showed downregulation with the highest repression in *T. erinaceum* bioprimed root (24 h) and leaf (48 h) tissue samples with 0.03 and 0.08 fold decrease, respectively. Qualitative expression of PR proteins (chitinases and glucanases) was found elicited in *T. erinaceum* primed plants. However, the antioxidative activity of tomato superoxide dismutase and catalase increased with the highest upregulation of *SOD* and *SlGPX1* in *Fol + T. erinaceum* treatments. We observed that these expression changes were accompanied by 32.06% lesser H_2_O_2_ production in *T. erinaceum* bioprimed samples. The aggravated defense response in all the treated conditions was also reflected by an increased lignified stem tissues. Overall, we conclude that *T. erinaceum* bio-priming modulated the defense transcriptome of tomato after the *Fol* challenged conditions, and were accompanied by enhanced accumulation of defense-related *WRKY* transcripts, increased antioxidative enzyme activities, and the reinforcements through a higher number of lignified cell layers.

## Introduction

Microbial bio-priming represents an adaptive strategy to improve the defensive capacity of plants that result in increased resistance/stress tolerance, and/or a more aggravated defense response against the stress challenged conditions. Plant growth-promoting fungi (PGPF) include many strains of *Trichoderma* spp., which have been used as a potential biocontrol agent. The rhizocompetent nature of *Trichoderma* spp. allows it to colonize roots, stimulates the plant immune system (induced systemic resistance; ISR), and pre-activation (priming) of the molecular mechanisms of defense against several potent phytopathogens (Hermosa et al., 2012; Pieterse et al., 2014; Martínez-Medina et al., 2017). Furthermore, colonization of this beneficial fungi promotes plant growth and also ameliorates the host plants against various abiotic and biotic stresses (Brotman et al., 2013; Zhang et al., 2016; Fu et al., 2017).

The WRKY family of transcription factors (Tfs) play a central role in plant development and the defense response against various abiotic and biotic stresses (Kiranmai et al., 2018; Singh et al., 2018; Vives-Peris et al., 2018) are plant-specific zinc finger type regulatory proteins. The WRKY proteins regulate the gene expression directly or indirectly by modulating the downstream target genes, by activating or repressing the other genes (encoding Tfs) or by self-regulating their own expression (Shankar et al., 2013). In tomato (*Solanum lycopersicum*), a total of 83 *WRKY* genes (previously documented 81 genes; Huang et al., 2012) has been identified (Bai et al., 2018; Karkute et al., 2018). WRKY TFs play an indispensable role in the regulation of diverse biological processes, but most notably are the key players in plant responses to biotic and abiotic stresses (Bai et al., 2018). The plant defense regulation involving WRKY proteins can be determined through dynamic changes in the levels of accumulated *WRKY* transcripts inside the cell (Chi et al., 2013). One of the most crucial aspects of *WRKY* gene regulation is that despite of having the functional diversity among different WRKY members, almost all analyzed WRKY proteins recognize and bind conserved TTGACC/T W-box sequences (de Pater et al., 1996; Rushton et al., 1996; Eulgem et al., 2000; Ciolkowski et al., 2008). However, DNA binding assays revealed the importance of the invariant “GAC” core consensus sequences of the core promoter element in a feasible DNA-protein interaction (Sun et al., 2003; Ciolkowski et al., 2008; Brand et al., 2010, 2013). Further, some WRKY members exhibit differences in their DNA binding preferences, which are partly dependent on additional adjacent DNA sequences lying outside of the TTGACY-core motif (Ciolkowski et al., 2008). By homology modeling, *in vitro* DNA-protein interaction-enzyme-linked immunosorbent assay and molecular simulation studies performed with different AtWRKY proteins revealed differences in DNA binding specificities (Brand et al., 2013). The two most important questions arise if all the WRKY factors bind with the W-box DNA; how the specificity for certain promoters is accomplished, and how the stimulus-specific responses are mediated by diverse members of the *WRKY* gene family. Later, studies revealed that besides the W-box-specific DNA binding, *WRKY* specific stimulus-response is facilitated by other essential components that regulate the biological function of different WRKY members (Brand et al., 2013). Furthermore, activation or repression through W-box and W-box like sequences is regulated at transcriptional, translational, and domain level (Phukan et al., 2016) and the interaction of WRKY Tfs with the W-box (with core motif TTGACC/T) and clustered W-boxes present in the promoters of downstream genes, regulate a dynamic web of signaling through the kinase or other phosphorylation cascades. Epigenetic, retrograde and proteasome-mediated regulation pathways enable *WRKYs* to accomplish a dynamic cellular homeostatic reprograming (Bakshi and Oelmüller, 2014; Phukan et al., 2016). Microbial priming is also associated with chromatin modification in promoters of WRKY Tfs genes that regulates SA dependent defenses, thereby promoting the enhanced expression of WRKYs upon pathogen challenged condition (Jaskiewicz et al., 2011). Furthermore, *WRKYs* role in transcriptional reprogramming becomes clearer because of *in silico* analysis of *cis*-acting DNA regulatory elements from the promoter region of stress-responsive *WRKYs* revealed the presence of various abiotic stress-responsive elements (Karkute et al., 2018). In this way, characterization of the promoter elements could help in understanding the functional dimension and regulation of WRKY members at the molecular level which is particularly important for those WRKYs that have been reported in the crosstalk between abiotic and biotic stress tolerance (Bai et al., 2018). Because of the tight regulation involved in the specific recognition and binding of WRKYs to the downstream promoters, they have been considered as a promising candidate for crop improvement (Karkute et al., 2018).

The rhizospheric microbiome (beneficial) are known to play an indispensable role in the transcriptional reprogramming required for plant defense against pathogens (Spence et al., 2014) and requires complex signaling cascades, involving multiple Tfs that primarily function as transcriptional regulators. At the molecular level, the biocontrol mechanism induced by *Trichoderma* is mediated through the adaptive recruitment and reprogramming of defense-related transcripts (Shaw et al., 2016). The transcriptional regulation of stress-inducible genes and the activation of an adaptive response (microbial symbiosis) are mediated and modulated through an immediate early expression of the *WRKY* genes (Chen et al., 2012). Therefore, the level of the WRKY proteins inside the cell accumulate sharply, which further directs the transcriptional regulation of the target genes through regulation with other *cis*-acting response elements (Chen et al., 2012). In addition, bio-priming with *Trichoderma* spp. has been found to be associated with the expression of several genes involved in regulating the general oxidative stress as well as osmoprotection. Further, the signaling cascade that regulates the plant pathogen interaction may involve classical phytohormones such as salicylic acid (SA), jasmonic acid (JA), and ethylene (ET) or may incorporate auxin, abscisic acid (ABA), cytokinins (CKs), and brassinosteroids (Robert-Seilaniantz et al., 2011). The pathogen-triggered immunity (PTI) and effector-triggered immunity (ETI) has been reported to function at differently regulatory levels and their signaling network is regulated by WRKY proteins (Shen et al., 2007; Mangelsen et al., 2008; Bakshi and Oelmüller, 2014). In one prominent example, it was found that in barley (*Hordeum vulgare*) *HvWRKY1* and *HvWRKY2* were found to activate by the FLG22 (a MAMP) and were reported to play a critical role by acting as a negative regulator (repressor) of the PTI against the powdery mildew fungus, *Blumeria graminis* f. sp. *hordei* (Bai et al., 2018).

*Trichoderma* induced bioprimed signaling involves a systemic resistance type immune response (Tucci et al., 2011; Yoshioka et al., 2012; Martínez-Medina et al., 2013), interconnected in a complex network of cross-communicating hormone pathways involving the JA/ET-induced systemic resistance (ISR) or SA dependent signaling mechanism. It has been documented that the early signaling events following the interaction of *Trichoderma* spp. with the host plant, is mediated through the pattern recognition receptors (PRRs) that activate the MAMPs/DAMPs-triggered immunity (MTI/DTI) (Hermosa et al., 2013; Ruocco et al., 2015). The WRKY mediated defense response regulates the signaling crosstalk through the activation of JA/ET-mediated signaling pathways, encoding repressors that suppress the SA regulated gene expression (Lai et al., 2008). Therefore, WRKY Tfs also acts as an important node of convergence between the SA and JA signaling (Pieterse et al., 2012). During pathogen challenged conditions, plants protect themselves through a plethora of mechanisms including activation of the ROS system, accumulation of H_2_O_2_, cellular reinforcement at the infection sites (through deposition of the suberin and lignin), expression of the plant pathogenesis-related (PR) proteins (Pusztahelyi et al., 2015) which further reflects the role of the SA mediated systemic acquired resistance (SAR) pathway (Gharbi et al., 2017). Among the differentially expressed PR proteins, chitinases and β-1, 3-glucanases are two major hydrolytic enzymes abundant in many plant species following the infection of different fungal pathogens (Ebrahim et al., 2011; Balasubramanian et al., 2012). Both chitinases and glucanases play a crucial role in plant defense against fungal pathogens since the cell wall of pathogenic fungi is composed of the two most crucial elements chitin and β-1, 3-glucan, and constitute the structural barrier of pathogenic cell wall fungi. Further, β-1, 3- glucanases appear to be coordinately expressed along with chitinases after fungal infection (Balasubramanian et al., 2012). The lignification event is an important mechanism which is accompanied by the accumulation of lignin or lignin-like phenolic compounds following the pathogenic attack and has been reported to occur in the plethora of plant-microbe interactions during the plant defense responses (Ebrahim et al., 2011). As a part of the host defense mechanism, lignification plays an indispensable role in preventing pathogen growth and dissemination. The lignified tissues represent the structural barrier and provide resistance in plants against biotic damages.

*Trichoderma* spp. induced bio-priming is characterized by the secretion of various antimicrobial compounds through the participation of the phenylpropanoid pathway that not only delimits the infection and dissemination of pathogens but also confers tolerance against various abiotic stresses (Mastouri et al., 2012; Ahmad et al., 2015). The inoculation of *Trichoderma* spp. leads to the activation of an efficient reactive oxygen species (ROS) detoxification system (De Palma et al., 2019). Further, mycoparasitic colonization by *Trichoderma* spp. leads into induction and accumulation of the PR proteins at early stages of root colonization (Yedidia et al., 2000). In many studies, it has been demonstrated that treatment of plants with beneficial microbes, particularly *Trichoderma*, enhances plant resistance against pathogens through ROS generation and lignification (Patel et al., 2017; Meshram et al., 2019).

In recent years, experimental studies done with *Arabidopsis thaliana* co-inoculated with *T. asperelloides* T203 provides sufficient data showing an increased expression of the specific *WRKY* genes and activation of the JA pathway that stimulates JA signaling through repression of the jasmonate ZIM domain (JAZ) repressors (Brotman et al., 2013). However, the signaling cascades and molecular events involved in *Trichoderma*-root association in the presence of elicitors or effectors from the pathogen *Fusarium oxysporum* f. sp. *lycopersici* (*Fol*) have not been investigated in light of *WRKYs* gene-mediated defense regulation of the host. Therefore, the objective of the present study is to provide a comparative approach for unraveling the *WRKY* gene-mediated defense signaling in the presence and absence of the beneficial microbe (*Trichoderma* spp.). The study was carried out to examine the real-time based relative quantification of differently expressed defense-related *WRKY* genes in tomato plants primed with *T. erinaceum* against the fungal pathogen *Fusarium oxysporum* f. sp. *lycopersici.* Additionally, we have also analyzed the time-dependent and tissue-specific expression profile changes in genes that constitute the ROS detoxification system, including superoxide dismutase (*SOD*), glutathione peroxidase (*GPX1*) and PR proteins. The biochemical response of the host has been investigated in terms of antioxidative enzyme activities, H_2_O_2_ content, and lignin deposition.

## Materials and Methods

### Fungal Inoculum Preparation

The pathogenic cultures *Fusarium oxysporum* f. sp. *lycopersici* (*Fol*) was brought from the Department of Mycology and Plant pathology, Institute of Agricultural Sciences, Banaras Hindu University (BHU). The fungal inoculum was prepared from the 7 day old culture of *Fol* pathogen as per the method suggested by (Taylor et al. (2013)). In brief, the Petri dish containing the culture was suspended with sterile distilled water. The spores were gently removed using a glass spreader, and then the heterogeneous suspension was filtered using muslin cloth for removing the mycelial mat. The filtered suspension was diluted with sterile distilled water to maintain a minimum density of 2 × 10^5^ to 2 × 10^6^ spores mL^–1^ as quantified through the hemocytometer.

### Pathogenicity Test

The pathogenicity test was performed with the *Fol* isolate to validate the Koch postulates for confirmation of the role of the pathogen in developing vascular wilt disease symptoms. The spore suspension of the *Fol* pathogen prepared above was used for pathogenicity testing. Twenty days old healthy tomato seedlings from both control and bioprimed plants were inoculated by the standard root dip method (Nirmaladevi et al., 2016). The healthy seedlings were uprooted from pots with gentle care without disturbing and disrupting the root integrity, shaken for removal of adhered soil particles and washed gently under the running tap water. The sterilized scissor was used for trimming the root apex portion (about 1 cm) and then the trimmed root portion was dipped in the prepared conidial suspension (2 × 10^5^ to 2 × 10^6^ spores mL^–1^) of the *Fol* pathogen for 30–40 min, for soaking in the conidial suspension. The inoculated seedlings were then used for transplantation to mini pots (15 cm diameter, surface sterilized with 0.1% mercuric chloride) containing the sterilized soil and sand mixed in a 2:1 ratio. Three seedlings per pot were used for transplantation. Plants were maintained in a greenhouse under 16 h light/8 h dark conditions with temperature ranging 28–29°C. Seedlings were watered on a daily basis. The symptoms of vascular wilt disease were initially observed 15–20 days after post inoculation of the *Fusarium oxysporum* f. sp. *lycopersici* (*Fol*) pathogen.

### *In vitro* Antagonistic Assay

Ten different isolates of *Trichoderma* spp. (T_1_–T_10_) were brought about from NBAIMCC, Kushmaur, Mau, Uttar Pradesh, India. The isolates were as follows: *Trichoderma fasiculatum* (T_1_) (NAIMCC-F-01714), *T. hamatum* (T_2_) (NAIMCC-F-01717), *T. koningii* (T_3_) (NAIMCC-F-01757), *T. longibrachiatum* (T_4_) (NAIMCC-F-01770), *T. pseudokoningii* (T_5_) (NAIMCC-F-01775), *T. asperellum* (T_6_) (NAIMCC-F-02170), *T. erinaceum* (T_7_) (NAIMCC-F-02171), *T. virense* (T_8_) (NAIMCC-F-02231), *T. piluliferum* (T_9_) (NAIMCC-F-02227), *T. viride* (T_10_) (NAIMCC-F-02500). The detailed information regarding the *Trichoderma* spp. used in this study including accession identities, source of isolation and isolation code has been provided in Supplementary Table S1. The *in vitro* antagonistic activity was checked for all the selected 10 isolates of *Trichoderma* spp. against the *Fol* pathogen, to find out the best isolate that could be used for seed bio-priming and further experiments. A 5 mm mycelial plug was cut with the help of cork borer from both the *Fol* Petri plate and each of the *Trichoderma* isolates (T_1_–T_10_) culture plates. The disks from *Fol* and each of the *Trichoderma* isolates were placed in a fresh culture plate with a separation of 3 cm apart from each other. The plates were then incubated at 27 ± 2°C for 5 days. The mycelial growth was recorded, and the percent inhibition was calculated. The experiment was done in replicates of three and the percentage inhibition of radial growth was measured from formula [100 × (C–T)/C] where C = radial growth of the pathogen in control and T = radial growth of the pathogen in dual culture with antagonist (Garrett, 1956).

### Preparation of *T. erinaceum* Spore Suspension and Seed Bio-Priming

The *Trichoderma* isolates showing the maximum growth inhibition (among the 10 isolates under study) of the *Fol* pathogen (*T. erinaceum*; NAIMCC-F-02171) was further grown on PDB medium for 7 days at 27 ± 2°C. The spores were harvested in sterile saline (NaCl 0.85%) and filtered with a sterile muslin cloth. The optical density OD was measured at 600 nm with OD of 1.026 that contained 2.26 × 10^7^ spores mL^–1^. The spore suspension was centrifuged at 10,000 rpm for 10 min. The pellet was re-suspended in the same volume of autoclaved 1.5% CMC (carboxy methyl cellulose) (Jain et al., 2012). The tomato seeds (S-22 variety) susceptible (83.67%) to the *Fol* pathogen (Chopada et al., 2014) were used in this experiment. For seed bio-priming with spore suspension of *T. erinaceum* the fresh and healthy seeds of tomato were surface sterilized with 0.01% aqueous solution of mercuric chloride followed by repeated washing with double-distilled water and further dried under laminar air flow on autoclaved blotting paper (Jain et al., 2012). The surface sterilized and dried seeds were treated by soaking in the spore suspensions of *T. erinaceum*. The control seeds were treated with only CMC without suspension. Further, all the seeds were placed in the moist chamber at 98 % relative humidity and 28–30°C and maintained for 24 h (Jensen et al., 2004).

### Pot Trials

The *T. erinaceum* bioprimed and control seeds were further sowed in the fresh plastic pots (having 08 cm diameter) containing the sterilized soil mixed with vermiculite (2:1). A total of 4 seeds per pot were sown for each treatment and a control set was maintained. The pots were irrigated manually on every alternate day. A total of five replicates for each treatment were prepared and maintained at 16 h light/8 h dark in greenhouse conditions with temperature 28–29°C and relative humidity in the range of 50–70% following the protocol (Zehra et al., 2017). All the seeds were allowed to grow in greenhouse conditions for 5–6 weeks. The 5–6 weeks old plants following the seed germination (height 15 cm) were treated with the *Fol* suspension following the protocol (Zehra et al., 2017). The root and leaf tissues from all the four treatments including control, *Fol* challenged, *T. erinaceum* bioprimed and the *T. erinaceum* bioprimed +*Fol* challenged were collected at the different time interval 0 h (control), 24 and 48 h for qRT-PCR analysis. Further, the biochemical assessment was done using the leaf tissues from all the samples collected at different time intervals.

### Morphological Growth Characteristic

The monitoring of the bioprimed plant and control (unprimed) plants was done regularly. The plants were first observed at 15 days and later on after 45 days interval for recording the characteristic changes observed in morphological growth parameters and other attributes such as increased plant height, root and shoot length, number of leaves, and thickness of the stem. The data were compared with the same day untreated control samples.

### RNA Extraction and cDNA Synthesis

The relative expression of distinctly upregulated *WRKY* transcripts under all the treated conditions were measured both quantitatively and semi-quantitatively in root and leaf tissues at different time intervals (0, 24, and 48 h), respectively. The total RNA was extracted using TRIZOL reagent (Invitrogen) following the manufacturer’s protocol. The 1.0% agarose gel (prepared in DEPC treated water) was used to check the quality of the extracted RNA. Further, the quantification, purity, and integrity of the extracted RNA were evaluated using Nanophotometer (Implen, CA, United States) at absorption ratio of 230/260/280 nm. The first strand of cDNA was synthesized through the iscript^TM^ cDNA synthesis kit (Bio-Rad Laboratories, United States) using 1.0 μg of the extracted RNA as per the given recommendation.

### Real-Time Quantitative PCR Analysis

The quantitative and semi quantitative studies were done for evaluating the spatial and temporal expression of accumulated *WRKY* transcripts as well as other defense related genes in all the four treatment conditions. Real-time quantitative PCR (qRT-PCR) reactions were performed using SsoFast^TM^ EvaGreen^®^ Supermix detection chemistry (Bio-Rad) with an iQ5 thermocycler (BioRad Laboratories, United States). The qPCR was performed in three independent biological replicates with each biological replicate performed in triplicates using the SYBR Green fluorescence dye (Qiagen, United States) and analyzed using iQ-SYBR Green Supermix (Bio-Rad, CA, United States) on iQ5 thermocycler (Bio-Rad, CA, United States) with iQ5 Optical System Software version 2.0 (Bio-Rad, CA, United States) following the protocols as mentioned. The PCR reactions were performed in a 20 μl final volume reaction mixture that contains 2 μl of the template cDNA (20 ng), 1 μl of each gene-specific primer (0.2 μM) and 10 μl of 2 × SsoFast^TM^ EvaGreen^®^ Supermix. The *WRKY* gene-specific primer was designed through the Primer 3 http://primer3.ut.ee/ (Untergasser et al., 2012) (Table 1). The protein sequences of glutathione peroxidase (SlGPX1; NP_001234567) and PR proteins including both PR2 (NP_001234158) and PR3 (XP_004237833.1) were used for designing gene specific primers. Further, sequences of all the primers were validated using Primer-Blast at https://www.ncbi.nlm.nih.gov/tools/primer-blast/ (Ye et al., 2012). The qRT PCR reaction program was set as an initial denaturation at 95°C for 10 min followed by 45 cycles of denaturation at 95°C for 15 s, annealing at 60°C for the 30 s and extension at 72°C for 30 s. The heat map was generated using the replicated count data Bio-conductor R^1^ obtained from expression values for both root and leaf tissues. The tomato *ACTIN* gene was used as a reference gene due to its constitutive and stable expression (Vergne et al., 2007). The Δ*C*_t_ value was calculated from the difference observed between the *C*_t_ values given for our target *WRKY* gene and the housekeeping *ACTIN* gene (that act as the constitutive control). The relative quantification was analyzed by using the 2-^ΔΔ*CT*^ method given by Livak and Schmittgen (2001) and then normalized to the C_t_ data about the transcript level of the *ACTIN* gene as an internal control because of its constitutive expression. The heat map was generated using Bioconductor R (see footnote 1) software tool.

**TABLE 1:** List of gene specific primers used for quantitative and semi-quantitative real time PCR studies.

**S.No**	**Gene name**	**Primer sequence (5-3′)**	***T*_m_**	**GC%**
1	*SlWRKY4* (Forward Primer)	CGTTGCACATACCCTGGATG	58.98	55.00
2	*SlWRKY4* (Reverse Primer)	GGCCTCCAAGTTGCAATCTC	59.19	55.00
3	*SlWRKY31* (Forward Primer)	CCACCTCCTTCACTTCCATT	57.11	50.00
4	*SlWRKY31* (Reverse Primer)	GATGGAAAACTCCCAGTCGT	57.53	50.00
5	*SlWRKY37* (Forward Primer)	CAGATGCAGCAGTTCAAAGG	57.37	50.00
6	*SlWRKY37* (Reverse Primer)	CTTCGAGGGACACATGTTGA	57.54	50.00
7	Chloroplast Cu/Zn-superoxide dismutase (SOD) (Forward Primer)	CTGGACTTCACGGGTTTCAT	57.81	50.00
8	Chloroplast Cu/Zn-superoxide dismutase (Reverse Primer)	TTTGGACCGGTCAATGGTAT	56.81	45.00
9	Chitinase (*CHI1*) (Forward)	GTCAAGGGGGACCTTGTTTT	60.20	50.00
10	Chitinase (*CHI1*) (Reverse	CATGTGTGACATGAGCGAAG	58.81	50.00
11	β-1,3-Glucanase (GNSL) (Forward)	AGACAACGTCCGAGGGTATG	59.18	55.00
12	β-1,3-Glucanase (GNSL) (Reverse)	TTTTTCAAGGGCCGAGTATG	56.03	45.00
13	Phospholipid hydroperoxide glutathione peroxidase (GPX1) (Forward)	ACCAGTTTGGTGGACAGGAG	60.00	55.00
14	Phospholipid hydroperoxide glutathione peroxidase (GPX1) (Reverse)	GCTGGAGAAGTGGTTGGAGA	60.39	55.00
15	Actin (Constitutive control) Forward primer	GAAATAGCATAAGATGGCAGACG	58.90	45.00
16	Actin (Reverse)	ATACCCACCATCACACCAGTAT	58.40	45.00

### Gene Prediction, Chromosomal Map, and *Cis*-Acting *DNA* Regulatory Element Analysis

We have predicted the location of three characterized *WRKY* genes (including *SlWRKY4*, *SlWRKY31*, and *SlWRKY37*) having clear-cut upregulation in all the *Fol* challenged tissues through the gene prediction tool. Further, the chromosomal location of each *WRKY* gene in the tomato genome was searched and a promoter scan was done to identify the putative *cis*-acting DNA regulatory elements including the position of the W-box DNA. For gene prediction, the topmost hit identifiers for each respective SlWRKY member having maximum query cover and percent identity values were selected (based on Blast-p annotation). The protein sequences of SlWRKY4 (XP_004235494.1), SlWRKY31 (NP_001306910.1; previously renamed as SlWRKY33A) and SlWRKY37 (NP_001308885.1) were collected from the NCBI. The CDS sequences were analyzed using BLASTx tool to check the full-length protein sequence. Further, the full-length protein sequences were searched using tBLASTn program of NCBI and was searched across the whole genome shotgun contigs (wgs) database with selecting organism name (*Solanum lycopersicum* taxid: 4081). The promoter region prior to translational start site was searched for each *SlWRKY* gene and was confirmed through Blastx tool [where TSS; represent the position of transcription start site (TATA-box position)] and polyadenylation (Poly A; Poly A represents the 3′ polyadenylation site) tail. The orientation of each *SlWRKY* gene in positive frame was determined through the Fgenesh^2^ server. The Gene display server (GSDS 2.0)^3^ (Hu et al., 2015) was used to map the position of promoter, coding sequences, intronic region along the length of each *SlWRKY* gene. Further, to locate the position of each characterized *SlWRKY* gene on tomato chromosome, the respective protein sequences were annotated using tBLASTn tool across the wgs contigs database. Further, the chromosomal location of each *WRKY* gene was retrieved from Ensembl-Blast tool. The Plant CARE promoter database http://bioinformatics.psb.ugent.be/webtools/plantcare/html/ (Lescot et al., 2002) was used for finding the *cis*-acting DNA regulatory elements that bind to the promoter region of the characterized *WRKY* genes.

### Biochemical Assessment of Plant Defense Response

#### H_2_O_2_ Quantification

The amounts of H_2_O_2_ produced and accumulated in leaf tissues collected from all the treated samples (control, the *Fol* challenged, *T. erinaceum* bioprimed and the *Fol*+ *T. erinaceum*) were quantified following the method as suggested by Jana and Choudhuri (1981). The leaves at different time intervals (0, 24, 48, and 72 h) were collected from all the treated samples. 200 mg of leaf tissue from each sample was crushed in 50 mM sodium phosphate (NaH_2_PO_4_) buffer (pH 6.5). The sample was then centrifuged at 8000 rpm for 20 min. The supernatant thus obtained was amended with 0.1% titanium sulfate (TiS_2_O_8_). The sample was again centrifuged and the intensity of the yellow colored solution was measured spectrophotometrically at 410 nm. The amount of H_2_O_2_ produced was calculated with an extinction coefficient of 0.28 μM^–1^cm^–1^ and was expressed as μmol g^–1^ fresh weight (FW).

### Assessment of Antioxidative Enzyme Activities

#### SOD Activity

The superoxide dismutase (SOD; EC 1.15.1.1) activity was measured following the method as suggested by Beauchamp and Fridovich (1971). 200 mg leaf tissues from all the treated leaf samples were crushed in a 5 mL extraction buffer that contained 0.1 M phosphate buffer (pH 7.5) and 0.5 mM EDTA. The samples were then centrifuged at 15,000 rpm for 15 minutes. The final assay mixture was prepared with a 50 mM phosphate buffer (pH 7.8), 13 mM methionine, 75 μM NBT, 60 μM riboflavin, 0.1 mM EDTA, 200 μl enzyme extract and 2 μM riboflavin was used for spectrophotometric calculations recorded at 560 nm.

#### CAT Activity

Catalase (CAT; EC: 1.11.1.6) activity was measured following the protocol as suggested by McKersie et al. (1990). The leaf tissues 200 mg from each of the treated samples was homogenized in a 5 ml of 50 mM Tris NaOH buffer (pH 8.0) that comprised of 0.5 mM EDTA, 2 % (w/v) PVP, 0.5% (v/v) Triton X-100. The absorption of the final assay mixture having 200 μL enzymic extract, 50 mM H_2_O_2_ and 100 mM phosphate buffer (pH 7.0) was recorded for 5 min at 240 nm and the activity was expressed as μmol of H_2_O_2_ oxidized min^–1^ mg^–1^ protein (extinction coefficient of 0.036 mM^–1^ cm^–1^).

### Histochemical Staining for Assessment of Lignification

The histochemical analysis for detection of the lignified tissue was done following the protocol as suggested by Guo et al. (2001). The transverse section (TS) of stem tissues from the second internode region was cut with Leica VT1000 Semiautomatic Vibrating Blade Microtome used for cutting the thin sections with thickness of 150 micron collected from all the treated tissues, and further, mounted in 1% phloroglucinol solution made with 95% alcohol. The mounted samples were covered with 0.1 mL concentrated HCl and was placed on a clean glass slide covered with the coverslip (Guo et al., 2001). The stained samples were visualized under a compound light microscope (Nikon, Japan). The lignified tissues were identified as intense pink coloration of deposited lignified material.

### Statistical Analysis

Statistical analysis was done using the statistical package SPSS (SPSS Inc., Version 16.0). All the experiments were performed in three independent biological replicates with each replicate performed in triplicates, and for statistical analysis, the mean value of each replication was used, one-way analysis of variance (ANOVA) performed for significance difference, while the mean separations were compared with Duncan’s multiple range test at the *P* ≤ 0.05 significance level.

## Results

### Pathogenicity Tests

**FIGURE 1:**
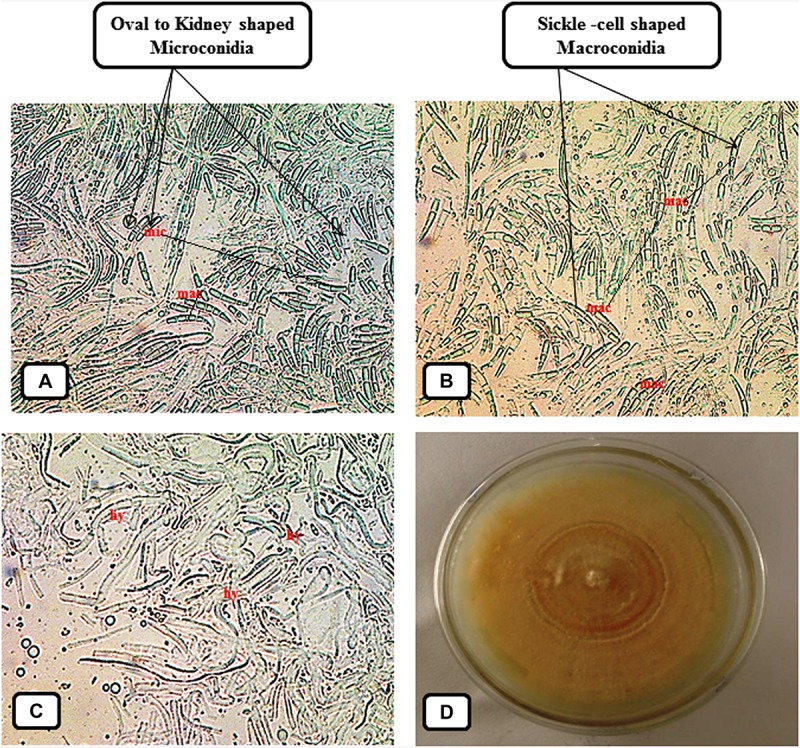
Photograph showing morphological and cultural characteristic of the vascular wilt pathogen *Fusarium oxysporum* f. sp. *lycopersici* (*Fol*) maintained on Potato Dextrose Agar (PDA) medium. **(A)** Microscopic observation of the small, oval or kidney shaped, and one or two celled, Microconidia (Abbreviation: mic) present in between the larger sickle shaped Macroconidia. **(B)** Microscopic view of the sickle-shaped, thin walled and delicate Macroconidia (Abbreviation: mac). **(C)** Structure of the fungal mycelium composed of interwoven hyphal filaments (Abbreviation: hy). **(D)** Photograph showing cultural characteristic and growth pattern of the *Fol* pathogen grown on Petri-plate.

The symptoms of vascular wilt disease were initially observed 15–20 days post inoculating the *Fol* pathogen. The earlier symptom developed was yellowing of the big and lower leaves followed by their drooping at later stages. At later stages, it was found that the upper aerial leaves had shown the loss of turgidity with dried lower leaves. On the basis of root-dip inoculation test (Nirmaladevi et al., 2016) pathogen was able to cause the vascular wilt disease, and was also recovered from the diseased wilted plants. In contrast, the uninoculated tomato seedlings did not develop any diseased symptoms, and therefore, validated the Koch’s postulates. The recovered *Fol* pathogen was grown in a fresh Petri-plate on PDA medium. The microscopic study was done to observe the structure of pathogenic hyphal filaments and conidial structure. The structure of fungal mycelium with oval or kidney shaped microconidia, and sickle shaped macroconidia has been shown (Figure 1).

### *In vitro* Antagonistic Assay

**FIGURE 2:**
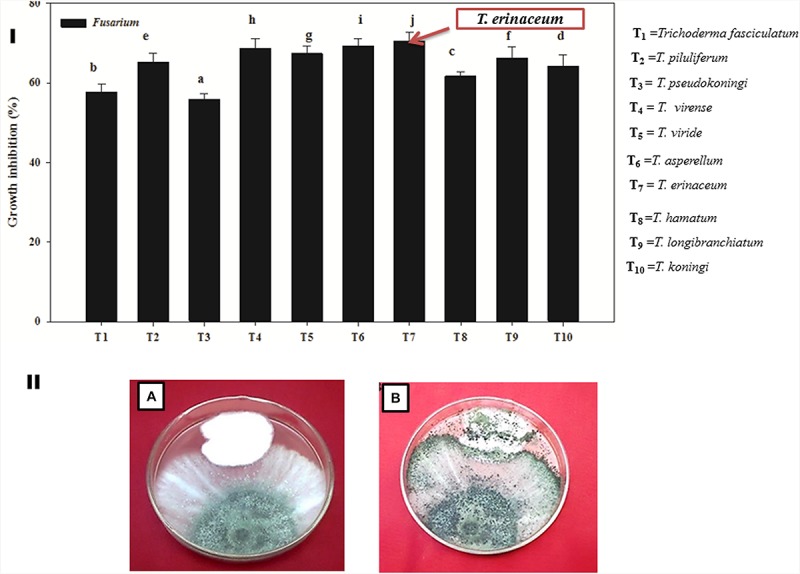
**(I)** Bar graph showing the efficacy of different species of biocontrol agent *Trichoderma* spp. in inhibiting the mycelial growth of the *Fol* pathogen. The figure represents the *in vitro* mycoparasitic interaction of different isolates of *Trichoderma* spp. (T_1_–T_10_) against the *Fol* pathogen through dual culture assay. The bar graphs represent the percentage growth inhibition of the pathogenic hyphae (*Fol*) by different species of *Trichoderma* employed in the study. The *T. erinaceum* (T_7_) showed the maximum inhibitory action against the *Fol* pathogen. **(II)** The Dual culture Assay showing the *in vitro* mycoparasitic and antagonistic activity of *Trichoderma* spp. against the pathogen *Fusarium oxysporum* f. sp. *lycopersici*. **(A)** Culture plate showing the mycoparasitic behavior of *Trichoderma* spp. and the *Fol* pathogen 5 days post inoculation. **(B)** The mycoparasitic activity of the *Trichoderma* showing the growth over the *Fol* pathogen and the picture was taken 10 days post inoculation of the *Trichoderma* spp. and the *Fol* pathogen (inoculated simultaneously). Mean (±SE) was calculated from three replicates for each treatment. Bars with distinct letters are significantly different at *P* ≤ 0.05 using the DMRT test.

It was found that all the isolates (T_1_–T_10_) of *Trichoderma* were found to be significantly effective in inhibiting the mycelial growth of the *Fol* pathogen over their respective control. However, *T. erinaceum* (T_7_) (Figure 2I) showed the best antagonistic activity against the *Fol* pathogen followed by *T. longibranchiatum* (T_9_) and *T. asperellum* (T_6_). Therefore, the *T. erinaceum* (T_7_) isolate was selected for seed bio-priming and greenhouse experiments. The dual culture assay showing the mycoparasitic interaction of *T. asperellum* with the *Fol* pathogen has been shown in Figure 2II.

### Morphological Growth Characteristic

**FIGURE 3:**
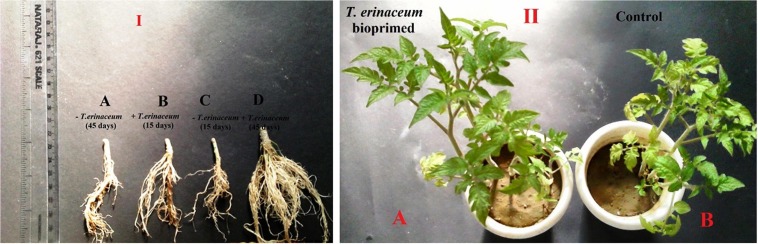
**(I)** Morphological growth characteristic of the *T. erinaceum* bioprimed and unprimed plants at two different intervals 15 and 45 days. The *T. erinaceum* bioprimed samples were found to have had profuse growth with increased plant height, root growth, stem diameter and number of leaves compared to control plants. **(A)** Picture showing root growth and thickness of stem at root-shoot junction after 45 days in the unprimed plants **(B)** *T. erinaceum* bioprimed plants after 15 days **(C)** *T. erinaceum* unprimed plants after 15 days **(D)** *T. erinaceum* bioprimed plants after 45 days. **(II)** **(A)** Pots showing the growth pattern and morphological parameters of unprimed (control) and *T. erinaceum* bioprimed plants.

The bio-priming of tomato plants with *T. erinaceum* resulted in the profuse growth of the tomato plants, bearing changes in several morphological attributes like an increase in plant height, the thickness of stem, root growth, root length, and number of leaves etc., and the same was compared with an untreated control sample. To observe the changes occurring in the morphological parameters, regular monitoring of the *T. erinaceum* bioprimed and unprimed (control) plants were done. The stem from *T. erinaceum* bioprimed and unprimed (control) were sectioned with a razor blade at the junction of root and stem to observe the morphological changes, particularly, measured in terms of root growth, and thickness of the stem collected at 15 and 45 day intervals and the same was compared with an unprimed (control) of the same day. The morphological changes in the root growth and the thickness of the stem in control plants compared with bioprimed plants collected at 15 days (Figure 3I-A) and 45 days (Figure 3I-B) have been shown. The photograph was taken to show the effect of the priming response of *T. erinaceum* on the morphological growth parameters of tomato plants (Figure 3II-A) and the unprimed plants (Figure 3II-B) has been shown.

### Gene Prediction, Chromosomal Map and *Cis*-Acting DNA Regulatory Element Analysis

Based on Blast annotation results we predicted the genomic position of each characterized *SlWRKY* gene that comprised of “TSS”, exonic coding sequences (both CDS_f_ and CDS_l_), and the “poly A” tail region across the whole tomato genome. The complete *SlWRKY31* gene coding sequence including TSS and poly A tail have been represented (Supplementary Figure S1A). The presence of TSS and poly A in the entire coding sequence, and particularly, the TSS before the poly A region revealed that our gene of interest is present in a positive frame, and the gene prediction result is accurate. The promoter region lying upstream of the translational start site of the *SlWRKY31* gene has been shown in Supplementary Figure S1B. The position of CDS encoding *SlWRKY4* including TSS and poly A tail has been shown (Supplementary Figures S2A,C). Further, the upstream sequences lying *SlWRKY4* and *SlWRKY37* have been shown in Supplementary Figures S2B,D. We identified a genomic locus of 8606 bp that contains the *SlWRKY4* gene, which is unambiguously mapped to a position on Chromosome (Chr) 3. Similarly, the genomic locus of 4065 and 3674 bp accommodated the *SlWRKY31* and *SlWRKY37* genes and were mapped on “Chr 6” and “Chr 1,” respectively. We found that besides W-box and other defense related elements, the promoter region of our characterized *SlWRKY* genes was flanked frequently with other abiotic stress-responsive elements like *HSE*, *ABRE* and *MBS* which clearly indicates their possible biological role in the management of abiotic stresses as well. The promoter region of tomato *SlWRKY4* and *SlWRKY37* genes have *LTR*, *ABRE*, *ERE*, *MYB* core elements. In contrast, the promoter region of tomato *SlWRKY31* gene was characterized by the presence of *TGA* motifs, *TCA element*, and *TATC box* along with other common motifs. The different promoters with their sequences and relevant functions have been shown in Supplementary Table S2.

### Gene Expression Analysis

**FIGURE 4:**
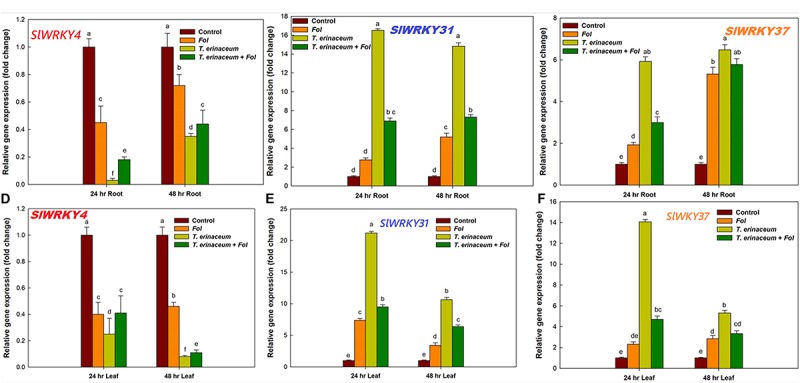
Quantitative PCR results showing the tissue specific and time bound differential expression of tomato *WRKY* genes in all the four defined conditions measured at different time intervals (0, 24, and 48 h). **(A)** The quantitative expression of *SlWRKY4* gene in root tissues data showing the relative change in expression values (fold change). **(B)** The relative fold change in expression value for *SlWRKY31* (previously named as *SlWRKY33A*; *SlDRW1)* expressed in tomato roots. **(C)** The relative fold change in expression value for *SlWRKY37* gene expressed in root tissues. **(D)** Relative fold change in expression values for *SlWRKY4* expressed in tomato leaves. **(E)** The expression data for *SlWRKY31* in leaves. **(F)** The expression data for *SlWRKY37* in leaf tissues. The *SlWRKY4* genes were found to be downregulated in both root and leaf tissues. Bars with distinct alphabetical letters are significantly different at *P* ≤ 0.05 applying the DMRT test.

The qRT-PCR studies unraveled the distinct temporal and the tissue-specific expression of tomato defense-related *WRKY* transcripts. In our previous study, we reported that during the *Fol* challenged conditions in tomato, a total of 16 different Sl*WRKY* genes were involved in plant defense, of which only three *WRKYs* (*SlWRKY4*, *SlWRKY31*, and *SlWRKY37*) were shown to have had clear-cut differential expression (Aamir et al., 2018). In the present study, we have measured the tissue-specific and time-dependent response, measured in the form of gene expression profile changes (Figure 4). The quantitative and semiquantitative expression of *WRKY*s were measured in terms of relative fold change in expression values compared to control tissues under the defined condition *Fol* treated (Aamir et al., 2018); *T. erinaceum* (bioprimed) and co-inoculated with both *Fol* + *T. erinaceum* challenged tissues, and the results were compared with unprimed (control) tissues. In the qPCR analysis, we found negative regulation (down regulation) of *SlWRKY4* in all the treatments in both root (Figure 4A) and leaf tissue samples (Figure 4D). However, the highest repression of the *SlWRKY4* was observed in root (24 h) and leaf (48 h) tissues with 0.03 and 0.08 fold decrease in *T. erinaceum* primed plants followed by *Fol*+ *T. erinaceum* treatments. In contrast, we found increased expression of *SlWRKY31* gene in *T. erinaceum* bioprimed plants (14.83 fold) in root tissues (at 48 h) followed by *Fol + T. erinaceum* treatments (Figure 4B). In leaf tissues, a similar trend of expression was recorded, however; upregulation in both genes was comparatively less than root tissues (Figure 4E). Interestingly, our results showed *Fol* + *T. erinaceum* bioprimed plants had an aggravated defense response as measured from the upregulated transcript profile of *SlWRKY31* and *SlWRKY37*. At early stages of treatments (0–24 h), we found increased expression of *SlWRKY31* and *SlWRKY37* in root tissues (Figure 4C). However, the expression of *SlWRKY31* in leaf tissues (at 0–24 h) was recorded to be comparatively less than roots. Further, one major contrasting difference recorded was that the expression of *SlWRKY37* in root tissue was less (0–24 h) than the leaf tissue of the same duration in *T. erinaceum* primed treatments. However, the expression in bioprimed samples further increases sharply in root tissues and decreases abruptly in leaf tissues (24–48 h). The *Fol*+*T. erinaceum* challenged samples showed the greatest change at 48 h in root tissue whereas a more or less similar expression trend was observed in leaf tissues (Figure 4F). However, in leaf tissues, the highest upregulation was observed in the transcript profile of *SlWRKY31* and *SlWRKY37* in *T. erinaceum* bioprimed tissues, explicitly, with 21.19 fold (*SlWRKY31*) and 14.07 fold (*SlWRKY37*) respectively. In this way, the results suggested that *SlWRKY31* and *SlWRKY37* function as a positive regulator and *SlWRKY4* as a negative regulator of plant defense programmed against the *Fol* challenged condition with a more aggressive defense response in *Trichoderma* pre-primed plants compared to non-primed plants.

**FIGURE 5:**
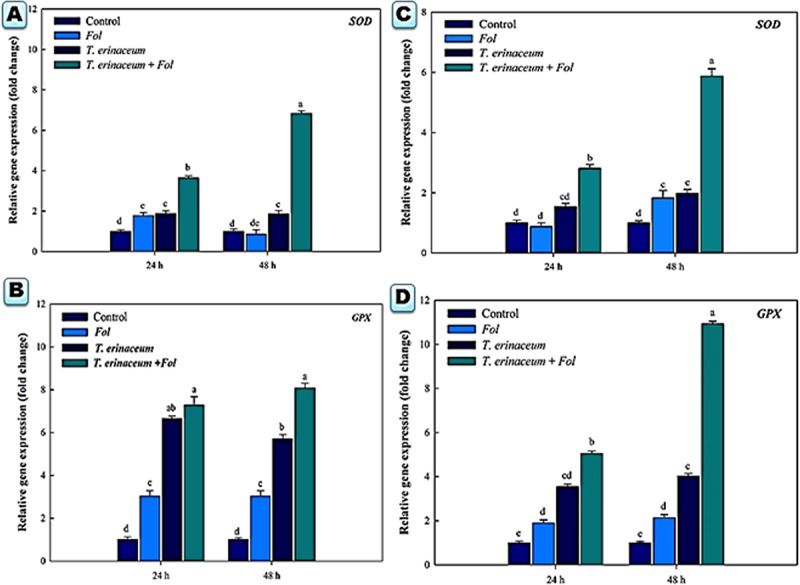
Quantitative PCR results showing the tissue specific differential expression profile changes of the Cu-Zn SOD and glutathione peroxidase encoding gene *SlGPX1* genes expressed in the root and leaf tissues and were measured at time intervals (0, 24, and 48 h) in each of the four different treatments. The data represents the relative fold changes in the expression values for the four different treatments. **(A)** The expression profile of the *SOD* gene in the root tissues. **(B)** The expression profile of the *SlGPX1* gene in the root tissues. **(C)** The gene expression profile of the *SOD* gene in leaf tissues. **(D)** The expression profile of the *SlGPX1* gene in leaf tissues. Bars with distinct alphabetical letters are significantly different at *P* ≤ 0.05 using the DMRT test.

**FIGURE 6:**
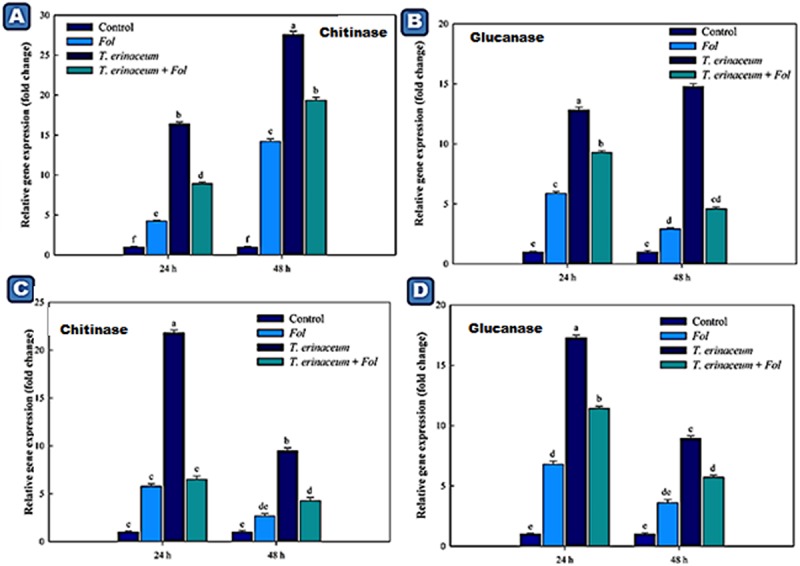
Quantitative PCR results showing the tissue specific differential expression of the genes encoding PR proteins (Chitinases and Glucanases) expressed in the root and leaf tissues and were measured at different time intervals (0, 24, and 48 h). The data represents the relative fold changes in the expression values for the four different treatments. **(A)** The expression profile of chitinase encoding gene (*CHI1*) expressed in the leaf tissues. **(B)** The expression profile of the glucanases encoding gene expressed in the leaf tissues. **(C)** The expression profile of the chitinase encoding gene in the root tissues. **(D)** The expression profile of the glucanase encoding gene in the root tissues and were measured at different time intervals. Bars with distinct alphabetical letters are significantly different at *P* ≤ 0.05 using the DMRT test.

We also checked the expression profile changes of the genes encoding transcripts involved in the cellular anti-oxidative defense mechanism. It was found that an upregulated expression of the *SOD* gene was recorded in *Fol* + *T. erinaceum* challenged root tissues (Figure 5A) compared to leaf tissues of the same time interval (Figure 5C). However, the expression of the *SOD* gene was more pronounced in *Fol*+ *T. erinaceum* treated plants, and highest expression with 6.84 fold (leaves) and 5.88 fold (root) increase was recorded in *Fol* +*T. erinaceum* challenged samples analyzed at 48 h. We found upregulated transcripts of *SlGPX1* in all treatments in root tissues measured at a different time interval (0, 24, and 48 h). However, the highest expression of *SlGPX1* was reported in the *Fol*+ *T. erinaceum* treated root tissues (Figure 5B) and to some extent in leaf tissues. Comparatively, *T. erinaceum* bioprimed leaf tissues were found to have more or less similar expression profiles at 24–48 h (Figure 5D). Further, the increased expression of PR-3 protein (chitinases) was found at initial hours (0–24 h) in *T. erinaceum* bioprimed followed by *Fol*+ *T. erinaceum* challenged root tissues (Figure 6A). In contrast, the highest upregulation transcript profile of chitinases in leaf tissues was recorded in *T. erinaceum* bioprimed samples with 16.39 and 27.57 fold increase at 24 and 48 h, respectively (Figure 6C). Further, an increasing trend similar to chitinases in expression profile of PR-2 (glucanases) was reported in *T. erinaceum* and *Fol*+*T. erinaceum* bioprimed root tissues (Figure 6B). In the case of leaf tissues, decreased expression of glucanases was recorded in all the tissue samples analyzed except *T. erinaceum* bioprimed leaf tissues (Figure 6D).

**FIGURE 7:**
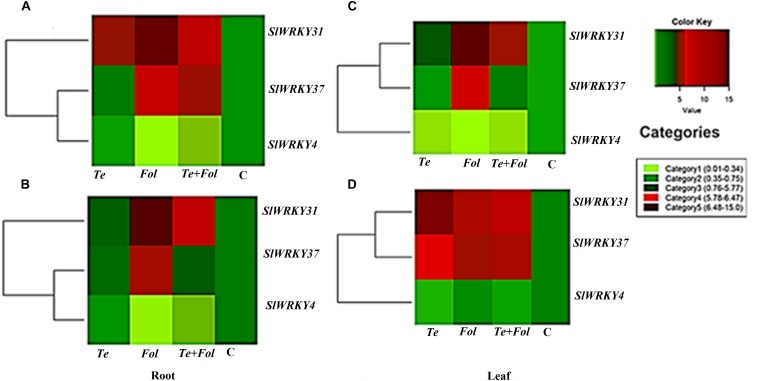
Heat map and clustering analysis of tomato *WRKY* genes expressed under the four different treatment conditions. The figure includes a “heat map” which is the part of the figure having different colors (red, green, and maroon) along with dendrogram. The rows represent genes while the treatments are shown as columns. The row dendrogram represents the gene clusters with similar pattern. The expression levels are mapped on the color scale provided at the right top of the figure along with the categories scale on the right. **(A)** The heat map showing the expression profile changes of the *SlWRKY4*, *SlWRKY31*, and the *SlWRKY37* genes in each of the four different treatments at 24 h in root tissues. **(B)** The heat map showing the expression profile changes of the *SlWRKY4*, *SlWRKY31*, and the *SlWRKY37* genes root tissues at 48 h. **(C)** The heat map showing the expression profile changes of the *SlWRKY4*, *SlWRKY31*, and the *SlWRKY37* genes in the leaf tissues at 24 h. **(D)** The heat map showing the expression profile changes of the *SlWRKY4*, *SlWRKY31*, and *SlWRKY37* genes in the leaf tissues at 48 h.

**FIGURE 8:**
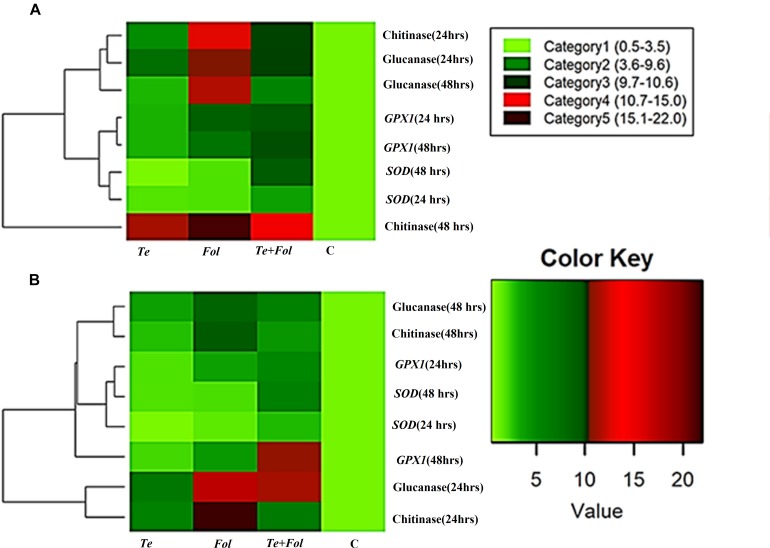
Heat map and clustering analysis of PR proteins and antioxidative defense genes in tomato. The rows represent the expression profile of defense related antioxidative genes and those encoding for PR proteins (chitinase and glucanase) that were expressed in a time-dependent and tissue-specific manner while the treatments are shown as columns. The row dendrogram represents the gene clusters with similar pattern. **(A)** The heat map showing the expression profile changes of the genes encoding for the PR proteins (chitinases and the glucanases) and antioxidative enzymes including *SOD* and *SlGPX1* in each of the four different treatments at 0, 24, and 48 h in the root tissues. **(B)** The heat map showing the expression profile changes at 0, 24, and 48 h in leaf tissues.

The semi-quantitative expression of each *WRKY* gene at different time intervals and in different tissue has been shown (Supplementary Figures S3A–D). The semi-quantitative expression of Cu/Zn-superoxide dismutase has been shown in Supplementary Figure S3E. The semi-quantitative expression of *SlGPX1*, Chitinase and β-1, 3 glucanases with respect to internal constitutive *ACTIN* gene have been shown (Supplementary Figures S4A–C). We have further analyzed the replicative count data for leaf tissue samples at 48 h through Bioconductor R for the *Fol* challenged, *T. erinaceum* bioprimed, *Fol*+ *T. erinaceum* bioprimed and unprimed control samples. The differential expression of the replicated count data was analyzed using the fold change expression values through Bioconductor R and have been represented in the form of heat map diagramme. The heat map diagramme for *WRKYs* expression profile changes in root tissue at different time intervals (0–48 h) has been shown (Figures 7A–D). The heat map diagramme for showing the relative expression values for *SOD*, *SlGPX1*, Chitinase and β-1, 3 glucanases in both root and leaf tissues for all the four defined conditions at different time intervals (24 and 48 h) has been shown in Figures 8A,B.

### Quantitative Estimation of H_2_O_2_

**FIGURE 9:**
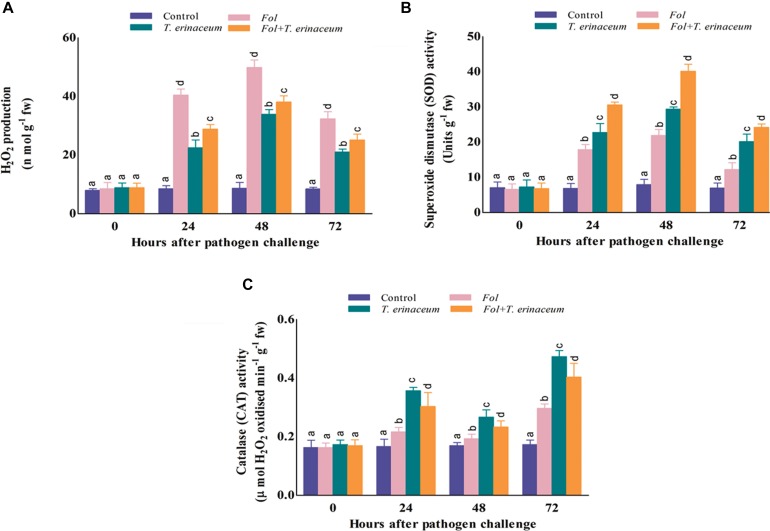
The comparative evaluation of biochemical activities of *T. erinaceum* bioprimed and unprimed samples under the *Fol* challenged conditions. **(A)** The H_2_O_2_ accumulated at different time intervals (0–72 h) in all the four different treatments. **(B)** Measurement of the SOD activity. **(C)** The CAT activity. Mean (±SE) was calculated from three replicates for each treatment. Bars with distinct alphabetical letters are significantly different at *P* ≤ 0.05 using the DMRT test.

The amount of H_2_O_2_ produced and accumulated was significantly higher in *Fol* challenged plants and was greatly reduced in the plants pretreated with *T. erinaceum* as compared to *Fol*+*T. erinaceum* treated samples. It was found that the amount of H_2_O_2_ produced was significantly higher at 48 h than recorded at early phases (0–24 h) of *Fol* inoculation. After 48 h post inoculation we did not find any further increase in the amount of H_2_O_2_ which confirmed the role of antioxidative defense enzymes under *Fol* challenged conditions. However, the amount of H_2_O_2_ generated and accumulated increased slowly in the leaves of all treatments but this rise was comparatively less than that observed in *Fol* + *T. erinaceum* treated samples (Figure 9A). Furthermore, it was found that the amount of H_2_O_2_ produced increased gradually from 0 to 24 h, was highest at 48 h and then declined successively in all the treatments. H_2_O_2_ content was 23.60% less in the *Fol* + *T. erinaceum* treated plants at 48 h when compared with *Fol* challenged plants. In *T. erinaceum* treated plants, it was 32.06% lesser than the *Fol* challenged plants.

### Assessment of Antioxidative Enzymes

#### SOD Activity

The SOD activity was found to increase successively on increasing time interval up to 0–72 h which declined thereafter. However, the SOD activity was reported to be maximum in case of the *Fol*+*T. erinaceum* samples at each time interval followed by the *T. erinaceum* bioprimed samples. The SOD activity was reported to be higher at 48 h post inoculation in all the pre-treated samples but found with maximum increase for the *Fol + T. erinaceum* bioprimed samples, followed by *T. erinaceum* and the *Fol* challenged condition, respectively (Figure 9B). Overall, the reported SOD activity calculated for the *Fol*+*T. erinaceum* challenged samples were 183.05% more than the *Fol* challenged leaf tissues, whereas in the case of *T. erinaceum* bioprimed tissues the calculated percentage increase in SOD activity was 133.94% higher than the *Fol* challenged leaf tissue samples.

#### CAT Activity

In our results, the CAT activity was found to be increased in the *Fol* challenged leaf tissues at the initial hour of *Fol* inoculation (0–24 h) compared to control (unprimed) samples. However, the CAT activity was at it’s maximum in *T. erinaceum* bioprimed samples in all the treatments followed by the *Fol* + *T. erinaceum* treated leaf tissues. One more interesting observation found that the CAT activity showed a fluctuating type pattern where it raised in the initial hour (0–24 h), decreased further, and then increased at 72 h post inoculation in all the treatments (Figure 9C). Furthermore, the rise in the CAT activity at later stages of treatment (48–72 h) was much more than those recorded for initial hour increment. In all the pre-treated tissues we found that the maximum rise in the CAT activity for *T. erinaceum* challenged plants 159.79% greater than the *Fol* challenged samples, followed by the *Fol* + *T. erinaceum* challenged samples. The CAT activity was comparatively 136.14% greater for the *Fol*+ *T. erinaceum* challenged (compared to the *Fol* treated leaf tissues). It has been well demonstrated that pre-treatment of biocontrol *Trichoderma* causes transcriptional reprogramming of the oxidative stress response, and accumulation of *ROS* gene network (SOD, CAT, GPx, APx) (Singh et al., 2011; Brotman et al., 2013) which results in increased activities of the antioxidant enzymatic pool.

### Histochemical Analysis for Lignin Assessment

**FIGURE 10:**
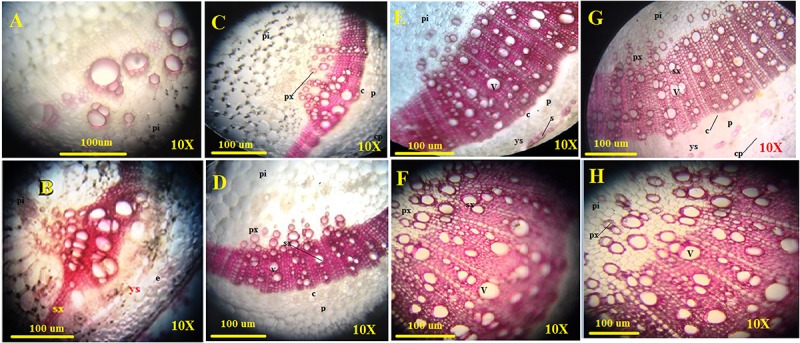
Assessment of the plant defense response in the form of lignification. The figure shows the transverse sections of stem tissues with lignin deposition in the walls of interfascicular fibers and xylem cells collected from four different transverse section of control, *Fol* challenged, *T. erinaceum* bioprimed, and *T. erinaceum* bioprimed + *Fol* challenged and were collected at two different intervals 15 and 45 days. The stem tissue sections from 2nd internode region were dissected through Vibratome, stained with Phloroglucinol-HCl (pink or fuchsia color). The intensity of the pink color represents higher deposition of lignin. Vascular bundles with lignified primary xylem; sclerenchyma starting to differentiate with no lignified cell walls. **(A)** Microscopic view of the histochemical section (transverse) of the control stem tissue taken from an unprimed plant (control) at 15 days interval. **(B)** The control stem tissue analyzed at 45 days interval. **(C)** The shoot tissue sections from the *Fol* challenged plant observed at 15 days interval. **(D)** The *Fol* challenged shoot tissues at 45 days interval. **(E)** The shoot tissues were taken from a *T. erinaceum* bioprimed plant at 15 days interval. **(F)** *T. erinaceum* bioprimed tissues at 45 days interval. **(G)** The shoot tissues taken from *Fol* challenged sample co-inoculated with *T. erinaceum* analyzed at 15 days interval. **(H)** The *Fol* challenged + *T. erinaceum s*hoot tissues analyzed after 45 days interval. All the images were observed at 10× magnification. Bar = 100 μM. px, primary xylem; sx, secondary xylem; f, xylem; pi, pith; p, phloem; c, cambium; v, vessel; cp, cortical parenchyma. The amount of lignin deposition was maximum in tissues that were *Fol* challenged and co-inoculated with *Trichoderma.*

The amount of lignified tissues was found to be greater in *T. erinaceum* plants co-inoculated with *Fol* (*Fol + T. erinaceum*) followed by *T. erinaceum* bioprimed samples. We have measured the deposited lignified tissues at two different time intervals i.e., on 15 days and later on, 45 days post inoculating the pathogen in both bioprimed and unprimed plants. The pink colored staining of Phloroglucinol-HCl stained lignified tissues, was more pronounced and was shown to have intense coloration. The *Fol* challenged sample had a comparatively higher amount of lignified tissue than uninoculated control as visualized under the compound microscope. The lignified tissue in the *Fol* challenged stem on 15 days (Figure 10C) showed less lignified tissues compared to stem tissues observed on 45 days (Figure 10D) whereas the control plant observed at 15 days (Figure 10A) and 45 days (Figure 10B) was found to have a comparatively lesser amount of lignified tissues. However, in all the histochemical tissue sections analyzed the deposited lignin was quantitatively more in *T. erinaceum* bio-primed tissue sections. The histochemical tissue sections from the *T. erinaceum* bioprimed plants at 15 days (Figure 10E) and 45 days (Figure 10F), respectively. The *Fol*+ *T. erinaceum* tissues were found to have comparatively more lignified tissues on 15 and 45 days, respectively (Figures 10G,H) which confirmed that the defense programming of the host was more pronounced in the presence of *T. erinaceum* induced microbial bio-priming under the *Fol* challenged conditions. Moreover, *T. erinaceum* pretreated plants co-inoculated with pathogen *Fol*+ *T. erinaceum* had the maximum amount of deposited lignified tissues which confirmed that the defense response in the presence of beneficial microbe (*T. erinaceum*) become more robust when plants further encounter the pathogen.

## Discussion

In their natural habitat, plants are continuously exposed to various abiotic and biotic stresses. However, plants adapt to such changes by acquiring a great degree of phenotypic plasticity that is mainly determined by the plant’s genome (Aamir et al., 2017). The integration of a multitude of partly synergistic and/or partly antagonistic signals enables plants to respond well against such extreme conditions (Pandey and Somssich, 2009). In many studies, it has been reported that members of the *WRKY* gene family contribute multiple biological processes involved in plant growth and development. Apart from this, the role of *WRKY* genes in the regulation of the stress response against the abiotic and biotic damages has also been demonstrated (Huang et al., 2012; Shankar et al., 2013; Bakshi and Oelmüller, 2014; Bai et al., 2018). Moreover, it has been reported that some WRKY Tfs function in combined stresses (both abiotic and biotic), and have been found active at crossroads of plant responses to both biotic and abiotic stresses (Qiu and Yu, 2009; Gao et al., 2011; Huang et al., 2012). For example, the tomato homologs (*SlWRKY31* and *SlWRKY33*) of *AtWRKY33* are activators of plant defense against several pathogens (Lippok et al., 2007; Liu et al., 2015; Li et al., 2016). In addition, the role of *SlWRKY31* and its homolog *SlWRKY33* was found to be involved in plant defense against drought and/or salt stresses (Huang et al., 2012). In tomato, many WRKY members have been demonstrated to act as a positive regulator of plant defense response against biotic stresses. Interestingly, different researchers redundantly annotated the tomato *WRKY* genes, and it is hard to follow the identical genes through different publications. For example, SlWRKY33 was published as SlWRKY33B and SlWRKY33A (Zhou et al., 2015) and SlWRKY31 was described as SlDRW1 (Liu et al., 2015). Likewise, *Arabidopsis* WRKY33 protein homolog in tomato SlWRKY31 (previously named as SlWRKY33A and SlWRKY33B; Zhou et al., 2015) was found to complement the function of the *atwrky33* mutant that was unable to provide tolerance against *B. cinerea* (Zheng et al., 2006). Overexpression of *S. pimpinellifolium* (closest wild relative of domestic tomato; The Tomato Genome Consortium, 2012) allele of *SlWRKY33* was found to provide resistance against hemi-biotrophic oomycetes *Phytophthora infestans* in tomato and *Phytophthora nicotianae* in tobacco. Similarly, in Group I, the closest homologs of *AtWRKY33* including *SlWRKY31* and *SlWRKY33* have been reported to provide defense against several soil-borne pathogens (Zheng et al., 2006; Lippok et al., 2007; Liu et al., 2015; Li et al., 2016). In contrast, the induction of *SlWRKY31* and *SlWRKY33* was observed under drought and/or salt stresses (Huang et al., 2012). In our previous report, we analyzed the Gene Expression Omnibus (GEO) datasets (GEO accession: GSE52336) to analyze the transcriptional profile of tomato defense-related *WRKY* genes. The bioinformatics analysis revealed that during *Fol* pathogenesis in tomato, a total 16 EST transcripts belonging to the *WRKY* gene family (based on Blast-x results and the presence of functional domain analysis) were found to be differentially expressed (Aamir et al., 2018). However, of these totally expressed transcripts, only three transcripts i.e., *SlWRKY4*, *SlWRKY31*, and *SlWRKY37* were found to have a clear-cut upregulated expression profile in all the *Fol* challenged samples (compared to control samples). The *in-silico* results were further confirmed through real time PCR and qRT-PCR studies. Apart from *WRKYs* role in plant-microbe interaction, Wenke et al. (2012) reported the role of the *WRKY* gene-mediated regulatory network in plant-biotic interaction, influenced through secretory volatile compounds (without having direct surface−to−surface contact) and that played, a significant role in plant’s fitness and vitality. De Palma et al. (2019) investigated the transcriptional response of tomato roots when colonized with endophytic *T*. *harzianum* T_22_. The root transcriptomes collected at different time intervals (0, 24, 48, and 72 h) post inoculation with beneficial fungus revealed the epigenetic and post-transcriptional regulation mechanisms (De Palma et al., 2019). *Arabidopsis* shares the highest evolutionary conservation with tomatoes (Yue et al., 2016) and the close phylogenetic relationship between tomato and *Arabidopsis* genes is well supported and exemplified by the fact that both species partially employ similar proteins for their developmental programming such as epidermal cell differentiation, development of root hairs, initiation of trichomes and accumulation of anthocyanins (Tominaga-Wada et al., 2013; Wada et al., 2014, 2015). The differential expression of tomato *SlWRKY2*, *SlWRKY3*, *SlWRKY4*, *SlWRKY6*, *SlWRKY7*, *SlWRKY23*, *SlWRKY51*, *SlWRKY53*, *SlWRKY80*, and *SlWRKY71* has been reported following the attack of *Cladosporium fulvum* in tomato plants (van Esse et al., 2009). In our results, we found that increased expression of the tomato *WRKY31* and *WRKY37* genes in the *Fol* challenged root and leaf tissues at increased time intervals (compared to control). Birkenbihl et al. (2012) demonstrated the role of *AtWRKY33* in the plant defense against the necrotrophic fungi *Alternaria brassicicola* and *Botrytis cinerea* through mutant studies. Later on, it was reported that *AtWRKY33* work as a global transcriptional regulator of metabolic and hormonal responses toward the necrotrophic pathogen, *Botrytis cinerea* (Birkenbihl et al., 2012). In a recent study, the time-dependent fluctuation in *WRKY*s gene expression was recorded following the attack of two pathogens *F. oxysporum* f. sp. *conglutinans* and *Pectobacterium carotovorum* sub. sp. *carotovorum* (Kayum et al., 2015). It was found that *Brassica rapa WRKY4* showed the highest expression on the 6^*th*^ day post inoculation of the pathogen. However, *P. carotovorum* subs. sp. *carotovorum* infection revealed fluctuation in *WRKYs* expression at different time intervals (Kayum et al., 2015).

We found the highest expression (compared to the *Fol* challenged samples) of *SlWRKY* genes in *T. erinaceum* pre-treated tissues followed by *Fol*+ *T. erinaceum* bioprimed tissue samples. In this context, Bakshi and Oelmüller (2014) reported that bio-priming with *Trichoderma* affects the expression of many defense-related genes involved in regulating ROS homeostasis and plant defense against stress response, particularly, *WRKY* genes. We found similar trends in our qPCR results, the highest upregulation was observed in the transcript profile of *SlWRKY31* with 16.53 fold (root) and 21.19 fold (leaves) increase in *T. erinaceum* bioprimed tissues at an initial hour (0–24 h) of treatments. These observations suggest that *Trichoderma* primed tissues reprogrammed the host defense machinery with respect to an elevated alarm state within tomato plants enrolling numerous *WRKYs* and other defense-related transcripts. In one report, the quantitative PCR (qRT-PCR) experiment when combined with *in-silico* results revealed the expression of defense-related genes in beans following the interaction of *T. velutinum* with *R. solani* or without *R. solani* (Mayo et al., 2016). It was found that of totally, expressed 48 genes, only *WRKY33*, *CH5b* (endochitinase precursor) and *hGS* (encoding for homoglutathione synthetase) showed upregulation in presence of *T. velutinum*. Further, it was found that treatment of *T. velutinum* resulted into downregulation of other genes or had no effect (*OSM34*) at all (Mayo et al., 2016). In contrast, *R. solani* infection caused downregulation of most of the genes analyzed, except *OSM34*, *CNGC2*, and *PR1* that were not affected. However, the presence of both *R. solani* and *T. velutinum*, showed downregulation of most of the genes analyzed, except upregulation of *hGS*, while other *CH5b* was not significantly affected (Mayo et al., 2016). In our study, we found downregulation of the *SlWRKY4* in both root and leaf tissues compared to upregulated *SlWRKY31* and *SlWRKY37* transcripts. However, the expression profile change for *SlWRKY4* was more conspicuous in root tissues at 24 h, which further decreased at 48 h.

The WRKY Tfs play an important role in the alleviation of both abiotic and biotic stresses (Bakshi and Oelmüller, 2014; Phukan et al., 2016; Bai et al., 2018). For example, *SlWRKY31* has been reported to offer defense against necrotrophic pathogens (Zheng et al., 2006) and also play a crucial role in drought and salt stress signaling (Huang et al., 2012). *SlWRKY3* provides resistance against salt stress and functions as a positive regulator against the root-knot nematode *Meloidogyne javanica* (Hichri et al., 2017; Chinnapandi et al., 2019). Similarly, downregulation of *SlWRKY4* has been reported in both abiotic (drought) and biotic stress (*Fol* pathogen) response (Aamir et al., 2018; Karkute et al., 2018). Therefore, identification and characterization of WRKY members that play dual function (managing both abiotic and biotic stresses) is an interesting approach and could be useful in crop improvement through plant breeding and/or transgenic technology. In addition, the functional relevance of WRKY proteins (having the dual function) and molecular mechanism of *WRKY* gene-mediated signaling at the point of multiple stresses is fully unexplored. Notably, *SlWRKY23* play a crucial role in the defense response against the powdery mildew fungus *Oidium neolycopersici.* However, *SlWRKY23* function under the saline stress condition when simultaneously challenged with pathogen was compromised rather than showing an additive effect (Kissoudis, 2016). With this view, we have analyzed *cis*-acting DNA regulatory element at the promoter region of characterized *SlWRKY* genes for finding their putative functions based on the presence of functional promoter elements. In a recent study, it was found that nine different *WRKY* genes in tomato showed upregulated expression under drought stress condition (Karkute et al., 2018). Further, *in silico cis*-acting DNA regulatory element analysis revealed that the promoter region of the characterized stress-responsive *WRKY* genes was flanked by various abiotic stress responsive elements like *ABRE*, *HSE*, *MBS* and Py-rich stretch, and were reported to promote high transcription levels (Karkute et al., 2018). We demonstrated the role of *SlWRKY31*, *SlWRKY37* and *SlWRKY4* in tomato defense response against the *Fol* challenged condition. However, promoter analysis results revealed the presence of different abiotic stress-responsive elements in *SlWRKY33* and *SlWRKY37* which showed their possible biological role in heat stress, drought stress response and hormonal response. In contrast, *SlWRKY4* could have a possible functional role in SA signaling and low-temperature response. In one report, Karkute et al. (2018) found that the promoter region of the drought stress-responsive *SlWRKY* genes were flanked by abiotic stress-responsive elements including *ABRE*, *HSE*, and *MBS*. 5’ UTR Py-rich stretch which supports our promoter search results. In our results, we found the same *cis* acting elements at the promoter region of *SlWRKY4*, *SlWRKY31* and S*lWRKY37* (encoding SlWRKY37 isoform I). It has been suggested that expression of the gene is governed by Tfs which binds to specific protein binding sites in the promoter region of the respective gene and therefore, characterization of the promoter element could provide critical knowledge about gene function and regulation (Karkute et al., 2018). The presence of W-box sequences recognized by *WRKY* proteins in the promoter region of heat tolerance related genes like *HSP* and *HSF* genes (*HSFA2*, *HSFB1*, HSP101, and *MBF1c*) further explains the function of *HSE* elements (Li et al., 2011). In this context, Fragkostefanakis et al. (2016) identified and characterized some heat stress-responsive genes in tomato male reproductive tissues that lack the functional HSE element in their promoter region and are regulated directly by WRKY Tfs which further confirms WRKYs role in the alleviation of heat stress response. Zhou et al. (2014) characterized tomato *SlWRKY33*, homologous to *AtWRKY33* in heat induced autophagy along with tomato autophagy-related (*ATG*) genes as it was found that silencing of *SlWRKY3*3 genes compromised tomato heat tolerance and reduced heat-induced *ATG* gene expression (Zhou et al., 2014). Overall, promoter analysis results revealed that our characterized *WRKYs* could have a possible biological role in managing both biotic and abiotic stress response.

The molecular mechanism underlying the manipulation of plant defense by beneficial symbiosis with *Trichoderma* spp. is still unknown. However, *Trichoderma*, promotes plant growth and development through several mechanisms including, root growth, increased nutrient uptake, and expression of plant defense-related genes (Mayo et al., 2016). Fungal interaction with plant roots could result in large-scale transcriptional re-programming events, in host tissues as well as fungus, and leads to transcriptional changes, therefore allowing the successful colonization of fungus to host tissues through transient repression of host immune responses (Morán-Diez et al., 2012; Brotman et al., 2013). In this context, Brotman et al. (2013) reported the increased expression of *A*t*WRKY40* and *AtWRKY18* after the colonization of *T. asperelloides* 203 to *Arabidopsis* root. Sáenz-Mata et al. (2014) reported the quantitative expression of 8 *WRKY* genes during its interaction with beneficial fungus *T. atroviridae.* The study concluded the existence of complex signaling route during *Trichoderma*-plant interaction involving both JA and SA signaling cascades and regulated by differential *WRKY* gene expression in *Arabidopsis* at the molecular level. Similarly, *T. harzianum* T_34_ colonization with *Arabidopsis* roots resulted into downregulation of defense-related genes and other Tfs including pathogenesis-related protein 1 (PR-1), *AtWRKY54*, flavin monooxygenase1 (*FMO1*), and glutathione transferases (Morán-Diez et al., 2012). Since *Trichoderma*, elicits induced systemic resistance (ISR) by the JA/ET-dependent pathway, and triggers a priming response in the plant (Korolev et al., 2008) the induced resistance provoked after a pathogen attack in unprimed tissues regulates through SA dependent signaling. Further, SA mediated signaling activates several jasmonate ZIM-domain genes and leads into downregulation of JA mediated responses. The final response of the signaling cascades results in the encoding of the JA responsive repressors along with other SA dependent WRKY Tfs, which causes susceptibility of mutant plants toward necrotrophic pathogens (Birkenbihl et al., 2012). This concludes into that *WRKY33*, a positive regulator of JA-related genes is a repressor of the SA pathway (Bakshi and Oelmüller, 2014).

The molecular mechanism that results in the modulation of defense transcriptome of plant tissues during *Trichoderma*-plant interaction is not fully explored. However, *Trichoderma* colonization to plant roots results into the secretion of secondary metabolites, proteins or other structural components that function as microbe associated molecular patterns (MAMPs). Further, plant-specific receptors use certain molecules (hydrolytic enzymes that may function on both pathogens and plant cell wall) could be employed as damage-associated molecular patterns (DAMPs) (Hermosa et al., 2013). These MAMPs and DAMPs stimulate the various signaling cascades, required for plant defense responses against phytopathogens, and mediated through multiple hormonal responses in cross-communication signaling pathways (Hermosa et al., 2013). In addition, plants have developed a plethora of defense strategies to combat pathogenic challenges. The most common mechanisms include the accumulation of ROS, the synthesis of PR proteins and phytoalexins, alteration in cell walls and enhanced activities of plant defense-related enzymes (Jothi and Babu, 2002; Bindschedler et al., 2006). Furthermore, the role of plant peroxidases (POs) has been well reported in defense responses including lignification, hypersensitive response, phenolics cross-linking and production of phytoalexins (Wojtaszek, 1997). The SAR regulated through SA signaling could result in direct activation and expression of the genes encoding PR proteins and in low dosages do not activate the defense genes in a direct way, but prime the tissues for potentiated defense-gene expression upon next *Fol* infection (Zehra et al., 2017). The *Fol* induced oxidative stress could be regulated by antioxidative defense enzymes including SOD and CAT that function along with other antioxidative enzymes to promote the oxidative damage by scavenging ROS (Saed-Moucheshi et al., 2014; Zehra et al., 2017). Moreover, *SOD* gene upregulation could result in diminished ROS activity and therefore higher accumulation of H_2_O_2_, and lead into the activation of the phenylpropanoid signaling. Additionally, the ROS intermediates play an important role in plant defense activation through cell-wall reinforcement, lignin biosynthesis, synthesis of secondary metabolites toxic to pathogen, activation of genes involved in plant defense, development of SAR against the targeted pathogen and/or exposure of the hypersensitive response (De Palma et al., 2016; Zehra et al., 2017).

In our results, root tissues had more expression of *SOD* gene compared to leaf tissues after the *Fol* infection at increasing time interval (0–48 h). Further, *T. erinaceum* bioprimed root tissues (0–48 h) were found to have an increased *SOD* expression compared to leaf tissues (24–48 h). In contrast, *Fol* + *T. erinaceum* treated leaf tissues showed a drastic change in *SOD* expression followed by the root tissues at later stages (24–48 h). Comparatively, it was found that in root tissues *SlGPX1* expression was more pronounced, and the *Fol*+ *T. erinaceum* treated root tissues showed the highest expression of *SlGPX1* than the leaf tissues of the same duration. In this context, Mastouri et al. (2012) reported an increased expression of the *SOD* gene after the tomato root colonization by *T. harzianum* T_22_, with a drastic rise in the relative expression of chloroplastic F-SOD_p_ and CZ-SOD_p_ but not cytosolic SOD_c_. Furthermore, it was found that roots maintained a relatively higher *SOD* activity under stress compared to control (declined *SOD* levels at later stages). The study concluded that T_22_ root colonization induced the *SOD* expression in chloroplasts and appreciably up-regulated the tomato *SOD* gene (Mastouri et al., 2012).

The biosynthesis of the lignin and other phenolic compounds are due to activation of the phenylpropanoid pathway (Patel et al., 2017; Zhou et al., 2018). Phloroglucinol-HCl staining (pink or fuchsia color) is a common method for lignin determination, and it is not a true lignin stain as it stains only cinnamaldehyde end-groups (Mitra and Loqué, 2014). Staining with Phloroglucinol-HCl yields a characteristic cherry pink or fuchsia color in the xylem and interfascicular fibers where these aldehyde groups are present. In our study, we found better illustrations of the lignified cellular layers at their early stages of development than at late stages because at late stages the clear-cut demarcation between the cells having actual lignified cellular layer might not be possible due to tissue differentiation. Lignin estimation through phloroglucinol staining specifically stains metaxylem and not sclerenchymatous tissues (Stafford, 1962). The tissue sectioning with Vibratome allows thin sectioning of equal thickness that assists in generating sharp images and reduces much the risk of producing inaccuracies observed due to differences in thickness. The core concept behind the thickness differences and image generation is that tissues having unequal thicknesses would transmit different intensities of light, and therefore, producing blurred images that generally occurred due to bad sample preparation. Although clear-cut demarcation of the observed differences between the lignified tissues are harder to visualize (Mitra and Loqué, 2014) even differences could be determined based on intake of pink coloration determining the amount of lignin deposited (variation in lignin content) by various tissue types.

## Conclusion

Microbial priming with *Trichoderma* spp. results into transcriptional regulation of defense-related genes with altered expression level. The modulated plant defense under primed condition is characterized by accumulation of defense-related transcripts and ROS molecules, activation of phenylpropanoid metabolism and accumulation of specific isoforms of hydrolytic enzymes such as chitinases and glucanases.

The differential tissue-specific and temporal expression of *WRKY* genes provided the evidence of diverse and complex signaling and transcriptional networks of plant response to beneficial interaction establishment. The highest expression of accumulated *WRKY* transcripts in *T. erinaceum* bioprimed tissues predicted a distinct signaling mechanism of the host (tomato) with *WRKY* genes, in the pre-stage bioprimed condition. Since the defense priming is clearly expressed at transcriptional level, research on mechanisms underlying the primed state has focussed on expression on signaling intermediates in transcriptional networks. Further, enhanced accumulation of *SlWRKY* transcripts in both bioprimed and *T. erinaceum* primed and co-inoculated with *Fol* pathogen plants revealed the transcriptional reprogramming of host defense during its colonization with *T. erinaceum* and during its antagonism/interaction made with the *Fol* pathogen. The data from our study provide sufficient evidence for the existence of a complex signaling network involving *WRKY* genes along with other signaling cascades during *T. erinaceum* induced bioprimed conditions. Further, bio-priming with *T. erinaceum* resulted into robust antioxidative enzyme profile changes, strong biochemical (accumulation of defense-related compounds) and structural changes (lignification).

## Author Contributions

MA conceived the idea, planned the experiments, performed all the experiments, did computational analysis of the results, and finally prepared and wrote the manuscript. VS assisted in computational analysis of the results. SK performed the quantitative and semi-quantitative PCR work. MD and AZ helped in some experimental sections, and also helped in writing, reviewing, and editing the manuscript. WA assisted in performing statistical calculations. RU and SS supervised the whole work. All authors read and approved the final version of manuscript for publication.

## Conflict of Interest Statement

The authors declare that the research was conducted in the absence of any commercial or financial relationships that could be construed as a potential conflict of interest.

## Footnotes

1 http://www.bioconductor.org

2 http://www.softberry.com/

3 http://gsds.cbi.pku.edu.cn

## Supplementary Material

FIGURE S1 **(A)** The position of CDS encoding *WRKY31* gene including the TSS and Poly A tail region as predicted through Fgenesh gene prediction tool. We have selected 1500 bp nucleotide upstream from the translational start site for promoter search for each *WRKY* gene. The CDSf represent the first type or First (Starting with Start codon), CDSi - internal (internal exon), CDSl - last coding segment, (ending with stop codon); TSS- represent the position of transcription start site (TATA-box position); Poly A represent the 3′ polyadenylation site. The presence of TSS before the first coding sequence CDSf and the Poly A tail after the last coding sequence (CDSl) predicted the complete coding sequence of *SlWRKY31* gene. Further, presence of TSS and Poly A in the entire coding sequence revealed that the gene of interest is in positive frame. **(B)** The structural organization of the *WRKY31*gene showing the intron-exon boundary and the upstream region with the position of CDS region that encodes each of the *SlWRKY31* transcription factor. The figure also show the other *cis-*regulatory element surrounding the promoter region of tomato *WRKY31* including the *TGA* and *TCA element.*

FIGURE S2 **(A)** The position of CDS encoded by *SlWRKY4* gene along with the TSS and the Poly A tail region. **(B)** The identified *cis*-acting regulatory elements (*ABRE*, *ERE*, *MYB*, and *LTR* elements) in the promoter region of the *SlWRKY4* gene with intron-exon boundaries including TSS (position of transcriptional start site; TATA box and poly A tail). **(C)** Position of the CDS encoded by the *SlWRKY37* including the TSS and the Poly A tail. **(D)** The location of different *cis*-regulatory elements surrounding the promoter region of the Sl*WRKY37* gene.

FIGURE S3 **(A)** The semi-quantitative expression profile changes in *SlWRKY4*, *SlWRKY31*, and *SlWRKY37* genes in four different treatments in both root and leaf tissues measured at different time intervals, and revealed through semi-quantitative PCR experiments. The expression of each gene was compared with a housekeeping *ACTIN* gene, having constitutive and stable expression pattern. **(A)** The expression of *SlWRKY4*, *SlWRKY31*, and *SlWRKY37* genes at 48 h interval in the leaf tissues. **(B)** The expression of *SlWRKY4*, *SlWRKY31* and *SlWRKY37* at 48 h interval in the root tissues. **(C)** The expression of the *SlWRKY4*, *SlWRKY31*, and *SlWRKY37* in the leaf tissues at 24 h. **(D)** The expression of the *SlWRKY4*, *SlWRKY33*, and *SlWRKY37* in the root tissues at 24 h. **(E)** The expression of the *CZn SOD* gene in both root and leaf tissues measured at different time intervals (0, 24, and 48 h).

FIGURE S4 The expression profile changes in genes that encodes for the tomato Glutathione peroxidase (SlGPX1), Chitinase and Glucanases in both root and leaf tissues at different time intervals, and revealed through the semi-quantitative PCR experiments. **(A)** The expression of the genes that encodes for GPX1 at 0, 24, and 48 h intervals in both root and leaf tissues. **(B)** The expression of the Chitinases in both leaf and root tissues at different time intervals. **(C)** The expression of the Glucanases in root and leaf tissues at 0, 24, and 48 h.

TABLE S1 List of different species of *Trichoderma* spp. used in this study along with their isolation source, isolate code and gene accession identity.

TABLE S2 List of different *cis* regulatory DNA elements that were present in the promoter region of *WRKY* genes in tomato along with their sequence and assigned functions.
