# Supplementary material for: Trichoderma erinaceum Bio-Priming Modulates the WRKYs Defense Programming in Tomato Against the Fusarium oxysporum f. sp. lycopersici (Fol) Challenged Condition
Source: Front Plant Sci. 2019 Jul 30;10:911. doi: 10.3389/fpls.2019.00911 (PMC6689972; doi:10.3389/fpls.2019.00911)
Supplement: FIGURE S1 — (A) The position of CDS encoding WRKY31 gene including the TSS and Poly A tail region as predicted through Fgenesh gene prediction tool. We have selected 1500 bp nucleotide upstream from the translational start site for promoter search for each WRKY gene. The CDSf represent the first type or First (Starting with Start codon), CDSi - internal (internal exon), CDSl - last coding segment, (ending with stop codon); TSS- represent the position of transcription start site (TATA-box position); Poly A represent the 3′ polyadenylation site. The presence of TSS before the first coding sequence CDSf and the Poly A tail after the last coding sequence (CDSl) predicted the complete coding sequence of SlWRKY31 gene. Further, presence of TSS and Poly A in the entire coding sequence revealed that the gene of interest is in positive frame. (B) The structural organization of the WRKY31gene showing the intron-exon boundary and the upstream region with the position of CDS region that encodes each of the SlWRKY31 transcription factor. The figure also show the other cis-regulatory element surrounding the promoter region of tomato WRKY31 including the TGA and TCA element. [file Data_Sheet_1.zip › 417677_Singh_Table_1.docx]

| S.No | | Name | Isolate Code | Accession  number | | Isolation  Source | |
| --- | --- | --- | --- | --- | --- | --- | --- |
| 1. | *Trichoderma fasciculatum* | **T_1_** | | NAIMCC-F-01714 | | NRCOG-TF-1, Onion rhizospheric soil, NRCOG, Pune | |
| 2. | *Trichoderma piluliferum* | **T_2_** | | NAIMCC-F-02227 | | Tri-2, Chickpea, M.K. Naik, Kasbe camp, Karnataka | |
| 3. | *Trichoderma pseudokoningi* | **T_3_** | | NAIMCC-F-01775 | | Rhizospheric soil of Cow pea, V Jayakumar, CARI Andaman & Nicobar Island. | |
| 4. | *Trichoderma virense* | **T_4_** | | NAIMCC-F-02231 | | Tri-15, Cotton, NRS, M.K. Naik, Shaktinagar, Karnataka | |
| 5. | *Trichoderma viride* | **T_5_** | | NAIMCC-F-02500 | | Tv-CARI-33, Black pepper, Someshwar Bhagat, Andaman and Nicobar Island | |
| 6. | *Trichoderma asperellum* | **T_6_** | | NAIMCC-F-02170 | | TWD1, rhizospheric soil, Krishna Kumar, Andaman and Nicobar Islands | |
| 7. | *Trichoderma erinaceum* | **T_7_** | | NAIMCC-F-02171 | | TND1, rhizospheric soil, Krishna Kumar, Andaman and Nicobar Islands | |
| 8. | *Trichoderma hamatum* | **T_8_** | | NAIMCC-F-01717 | | TNL-2, rhizospheric soil of *Lagenaria sinceraria* CARI Andaman & Nicobar Island. | |
| 9. | *Trichoderma longibrachiatum* | **T_9_** | | NAIMCC-F-01770 | | CABI-352545, Pitchvaram India | |
| 10. | *Trichoderma koningii* | **T_10_** | | NAIMCC-F-01757 | | CABI-299898, Agricultural waste, Lakshmikant, India | |
